# Attitude, Knowledge, and Practices of Bahraini Parents Regarding Children’s Oral Health and Early Childhood Caries: A Qualitative Study

**DOI:** 10.7759/cureus.95718

**Published:** 2025-10-29

**Authors:** Eman Alrowaili

**Affiliations:** 1 Oral and Dental Health Services, Primary Healthcare Centres, Manama, BHR

**Keywords:** added sugars, carbonated and sugary drinks, cultural and social factors, early childhood caries, middle eastern, oral hygiene practice, parents’ knowledge and practices, qualitative research, topical fluoride

## Abstract

Background: The high prevalence of caries in children in Bahrain necessitates understanding the parental factors that influence young children’s oral health, as they depend on their parents for diet and oral hygiene. The study aims to explore parents’ attitudes, knowledge, and practices, and the factors that influence them in relation to children’s oral health, especially early childhood caries.

Method: A qualitative study using face-to-face interviews and semi-structured open-ended questions was conducted on a sample of 12 parents recruited from a Pediatric dental clinic. Data was analyzed using thematic analysis.

Results: The parents included in the study cared about their children’s oral health in general. However, they had insufficient knowledge about fluoridated toothpastes and the cariogenicity of many dietary items, and the exfoliation time of primary teeth. In regard to practices, there was early introduction to bottle-feeding and late weaning, and early introduction to free sugars, especially sweetened drinks. Parents in general introduced oral hygiene practices early to their children, but mostly used toothpaste with insufficient fluoride concentration and irregular supervision of brushing. All these factors contributed to early childhood caries. Multiple environmental, social, commercial, and cultural factors affected parents’ choices and practices related to children’s oral health, such as living in joint families, fear of depriving children, and buying sugary items in bulk. Pain was the main instigator for seeking treatment and changing dietary and hygiene habits. Parents were open to early prevention and recognized and recommended opportunities for the same.

Conclusion: Parents mostly had a positive attitude towards children’s oral health at a young age. Their knowledge about oral hygiene practices, the use of fluoridated toothpaste, early diet, and dietary items with free sugars needs to be improved. Parents’ practices related to children’s oral care and diet starting at an early age are affected by multiple environmental and cultural factors.

## Introduction

Dental caries is the most common noncommunicable disease and is highly prevalent in childhood. Risk factors for childhood caries include early introduction of sugar before 24 months of age, late weaning, lack of knowledge about fluorides [1], going to sleep with a bottle, frequent consumption of sugars, and insufficient oral hygiene [2]. These factors determine children’s future preferences and habits and are affected by parents’ attitudes, beliefs, knowledge, and practices [1]. This is of concern, especially for preschool children, who depend on their parents for both diet and hygiene [3]. Parents’ perceptions and practices, in turn, are affected by multiple social, economic, cultural, and environmental factors [2]. 

Most studies on pediatric dentistry are quantitative and may not represent parents’ perceptions [4]. A deeper understanding of such perceptions and what influences them can be attained through qualitative studies [3] as they are flexible and allow an open scope of knowledge [5]. For example, several qualitative studies from different regions discussed factors related to parents’ attitudes and knowledge about caries [6], knowledge about the cariogenicity of daily consumed items [7], and the barriers parents face in maintaining their children’s oral health care routine [8,9].

Considering parents’ perceptions and the factors that affect their choices is important in setting preventive and promotional programs that take into account the local situation and culture [9]. This is important, knowing that the population in Bahrain is young, with 18.3% of the total population being 0-14 years of age [10], and caries prevalence in 2015 among six-year-olds was 86.8% [11]. The need for early and effective caries preventive programs for children, particularly early childhood caries, necessitates studying the varied factors that play a role in childhood caries in the population, one of which is parental factors. The study aims to understand Bahraini parents’ knowledge and daily practices around children’s oral health, and how culture and environment influence them, as early as infants’ feeding choices and early hygiene practices. Qualitative studies that reflect this knowledge about Arab parents are few, and this will be the first qualitative research in Bahrain and in Primary Healthcare Centres (PHCC) to assess parental factors related to childhood caries.

This article was previously posted to the Research Square preprint server on March 4, 2025 [12].

## Materials and methods

The study was conducted in Hamad Town Health Centre, Primary Healthcare Centres, Northern Governorate, Bahrain, that covers referrals from dentists in the northern governorate health centers (HC) for uncooperative children up to 12 years of age. Ethical approval for the study was obtained from the Research Committee of Primary Healthcare Centres. The theoretical framework of the study is based on realistic epistemology with an inductive qualitative descriptive approach [13,14]. Qualitative description studies aid in learning from participants’ experiences, which is important for policy recommendations that aid in developing effective and culturally sensitive interventions [13].

Participants’ selection and sampling

Participants were conveniently selected from among parents who came for their children’s dental treatment in the aforementioned pediatric clinic, with the exclusion of those parents who had a special needs child. Parents were approached to participate in a face-to-face semi-structured interview by the researcher. For transparency, the researcher explained the research, its aims, and method to each parent before taking written consent. Interviews were conducted until saturation of the data was reached, when new interviews did not provide different information, or added codes or themes to what was gathered from previous ones. Based on that, interviews were stopped after the 12th interview.

Researcher and relation with participants

The researcher had been practicing dentistry for around 24 years and had worked most of that period in the northern governorate. For more than a decade, she worked in school dental clinics and pediatric clinics. Being a clinician-researcher allows for a deep understanding of issues related to patients [15], and in this study, it allows for understanding caretakers’ issues related to children’s oral health. To allow building a rapport with participants, the interview, when possible, was conducted at the second visit.

Data collection

Face-to-face interviews help explore perceptions and experiences in depth and detail [16] and allow for reaching new concepts and achieving richness of data [13]. The interviews were conducted between June and October 2023 in the clinic using semi-structured and open-ended questions and probes that were formulated based on literature, experience, and observations of the researcher (see Appendices). It ensured consistency between all interviews but allowed flexibility. The questions were piloted a few times with parents not included in the study, without recording, and once with recording to gauge the interviewer’s interaction, since she would provide diet and oral hygiene advice at the end of the interview.

The recording was done using a smartphone secured with a passcode. No field notes were taken so as not to distract the participant and interrupt the flow of the interview. The interview took around 15-20 minutes, followed by 10-15 minutes of oral hygiene advice and diet analysis and advice. This allowed further discussion and openness, especially as the researcher was realistic in giving diet options and showed empathy with participants when they voiced difficulties and barriers. Interviews continued till data saturation was reached, as no new information or new codes, themes, or subthemes were generated. No repeat interviews were taken, especially after oral health education and diet advice were given, as that might affect the participants’ answers. Transcripts were not transferred to participants for comments, as the value of this step is doubtful and might be embarrassing and stressful to participants [17].

Data analysis

Thematic analysis was used [14,18]. It is based on sound theoretical and methodological methods and is flexible and widely used [14]. Anonymity of the participants was maintained from the time of the recording, using the Arabic method of respectfully calling a parent (mother of or father of <child’s name>). Later, codes and numbers were used while transcribing. The researcher conducted the interviews, transcribed them verbatim manually, and again read them for analysis; hence, she was familiar with the data. Coding and analysis were done manually. Open, line-by-line coding [18] was started with the first few interviews. Initial codes were generated, then as new interviews were taken and transcribed, codes were constantly modified, reviewed, and aggregated to create initial themes. Those were reviewed later to create final themes and subthemes. Minor themes were recognized and grouped where seen fit. For reporting, only the relevant participant statements were translated from Arabic to English. To ensure quality of reporting, the COREQ (COnsolidated criteria for REporting Qualitative research) checklist was used [15].

## Results

Thirteen parents were approached, and only one mother declined, citing being uncomfortable with recording. The 12 participants consisted of eight mothers and four fathers; their ages ranged from 25 to 47 years, and six of them were employed. The age of the youngest child in all the families ranged from six months to nine years. Other general quantitative characteristics of the participants are presented in Table 1.

**Table 1:** General characteristics of participants

Characteristics	Mean	SD
Age of parent in years	35.7	7.4
Number of children in the family	4.5	3.0
Age (in years) of child receiving dental treatment	5.5	2.7
Age (in years) of youngest child in the family	2.7	2.6

The qualitative analysis produced four main themes, namely knowledge, attitude, and practices related to (i) primary teeth, (ii) children’s diet, (iii) childhood caries, and (iv) sources of information, and multiple subthemes. These are presented in Table 2. Corresponding parents’ quotes are presented in Table 3.

**Table 2:** List of themes and subthemes

Themes	Subthemes	
I) Knowledge, attitude and practices related to primary teeth	1.1 Importance, eruption and exfoliation	
	1.2 Knowledge, attitude and practices related to children’s oral hygiene	1.2.1. Age of starting oral hygiene and regularity
		1.2.2. Use of toothpastes
		1.2.3. Practices related to toothpaste amount, supervision, and spitting
		1.2.4. Challenges and efforts
II) Knowledge, attitude and practices related to children’s diet	2.1 Infants' diet	
	2.2 General diet	
	2.3 Consumption of sugary foods and drinks	
	2.4 Factors affecting children’s intake of free sugars	2.4.1. Surrounding environment and commercial factors
		2.4.2. Large family / family circumstances
		2.4.3. Differences between parents
		2.4.4. Buying sugary items in bulk
		2.4.5. Child’s nature
		2.4.6. Fear of depriving children
III) Knowledge, attitude and practices related to childhood caries	3.1 Perceptions about cause of caries	
	3.2 Reason for child’s first dental visit	3.2.1. Pain
		3.2.2. Effect of decay on child and family
	3.3 Parents’ efforts to prevent and control childhood caries	3.3.1. Changing habits
		3.3.2. Inquiries about fluoride varnish
		3.3.3. Parents’ suggestions for improving services
IV) Sources of information		

**Table 3:** Parents' quotes

Quote number	Quote
1	“From the time she started teething, I started using the brush that looks like rubber and then as she got more teeth, I changed the brush to ones with more bristles.”
2	“We know from the ads that toothpastes have substances that are good for teeth. Children’s toothpaste packaging attracts children and the taste is milder.”
3	“When my daughter was around three years old, I used child toothpaste for her because it had the age range written on it. I thought I could not use our toothpaste for her. If someone does not know the- correct- information, it is easy to get deceived with what is available in the market.”
4	“Kid’s toothpastes are safer if they swallow it.”
5	“His mother puts little toothpaste for him. Sometimes he sees me and puts little toothpaste for himself, sometimes he fills the toothbrush.”
6	“My son is very stubborn, and he does not agree (to brush). Sometimes I shout at him. Yes, sometimes he opens his mouth, sometimes he does not, so I tell him he will not go to the cold store, or ride in the car or go anywhere, so he starts brushing again.”
7	“I knew my children felt full when I breastfeed them, so why would I introduce a bottle […] My daughters also breastfeed their infants […] and when you see the child, you can notice their good weight.”
8	“There is no time to breastfeed all the time […] I feel she - gets hungrier after she became five months old, she needs more nutrition, and more time but I have other children to care for.”
9	“I do not like pacifiers because they fall to the ground […] then another child picks it up and puts it in his or her mouth. Sometimes hair might get wound around it.”
10	“My children eat items from the cold store [….] they do not eat homemade food, but he – son’s name- sometimes drinks milk.”
11	“Making hospital food”
12	“My children drink juices, not juice drinks.”
13	“My children drink juice with every meal, and if there’s no juice they won’t eat.”
14	“I do not think of juices in term of their sugars content, I look at them as liquids. The problem is that my children forget to drink water […] that is why I find it easier that they drink juice.”
15	“He asks me why I give him a small size juice packet (150 ml); he wants the bigger one, he says ‘I am not a small child now’.”
16	“You do not get healthy options at the cold store.”
17	“They place these large colorful lollipops at the cashier, and […] children get attracted to them and then they want them. They should place them somewhere else.”
18	“We live with my husband’s family. My daughter meets with her cousins every day […] their mother is a nurse, and she brings them every day, and they bring sweets with them. Their aunt and their grandmother also give them sweets. My husband says I should not say no if they give our daughter anything.”
19	“My parents do not like children to eat the stuff sold at the cold store, but their father’s side are different; they say let the children go to the cold store and buy what they want.”
20	“Bringing the cold store home”
21	“Chocolate sandwich, cupcake, juice and popcorn.”
22	“My son sometimes says I am a bad mother because I do not allow him to buy all the snacks he wants.”
23	“Parents might give in and buy what their children see, so they do not feel deprived.”
24	“Children look at each other in school. I try to explain to my son that different people might have different options, some more than the rest. But if I tell him ‘No’, his achievement at school gets affected. It happened before and I do not want that.”
25	“My daughter had decay because of eating sweets, she had also taken iron supplements […] and she would sleep with the bottle in her mouth.”
26	“It was tiring […] my son getting up at night with pain and would not go back to sleep.”
27	“Yes, teeth ache affected her. My daughter was terribly upset, she did not want to do anything, and that upset us as well.”
28	“We used to buy Packets of sweets and snacks and put them on the table but after my daughter developed decay (and had pain), we stopped.”
29	“After my daughter had teeth pain, she accepted the changes we made for her diet […] even my other daughter after seeing her sister suffering with pain accepted the changes we made.”
30	“I saw ads about the protection layer. I saw that it is placed on teeth early to prevent decay.”
31	“We have children’s appointment at the MCH, why not allocate something similar for the child with the dentist [...] to give us advice on what to do, or find if the child has decay and then treat it from the beginning.”
32	“I stopped buying children’s toothpaste, but you know how social media is, it makes me doubt myself.”

Knowledge, attitude, and practices related to primary teeth

Importance, Eruption, and Exfoliation

The role of primary teeth for eating and chewing was clear, and some parents recognized the need for early treatment of primary teeth to prevent complications, but the nature of such complications was not clear, especially for future arrangement of permanent teeth. Knowledge about the eruption and exfoliation of primary teeth and the eruption of permanent teeth was linked to parents’ experience with their children.

Knowledge, Attitude, and Practices Related to Children’s Oral Hygiene

For most parents, brushing children’s teeth for cleanliness was the norm. 

Age of starting oral hygiene and regularity: Most parents introduced oral hygiene practices to children at around 18 months to two years of age and reported regular tooth brushing. Parents started with varied oral hygiene methods, wiping toddlers’ teeth, using small thumb-sized brushes, or with a toothbrush and water alone. A mother who started oral hygiene early with her daughter explained the methods she used:

“From the time she started teething, I started using the brush that looks like rubber and then as she got more teeth, I changed the brush to ones with more bristles.”

Parents who started oral hygiene late did so at their children’s enrollment in kindergarten or school and reported irregularity in brushing. Some parents noticed that their children were influenced to brush after seeing them or their sibling brushing.

Use of toothpaste: Even though some parents introduced an oral hygiene routine early, they deemed children too young to start using toothpaste and delayed it till children were older than two years of age. Commercial advertisements were mentioned to have played a role in the knowledge about toothpastes. Some parents thought the more costly the toothpaste, the better, and most of them used variants of children’s toothpaste as they had appealing packages, and the age range was indicated on toothpaste packages. A mother commented about the benefit of toothpastes in general and the reasons children prefer toothpastes that are marketed to them:

*“We know from the ads that toothpastes have substances that are good for teeth. Children’s toothpaste packaging attracts children and the taste is milder.”* 

Another mother explained her reasons for choosing a children’s toothpaste variety for her daughter:

“When my daughter was around three years old, I used child toothpaste for her because it had the age range written on it. I thought I could not use our toothpaste for her. If someone does not know the- correct- information, it is easy to get deceived with what is available in the market.”

Few parents had knowledge about fluoride presence in toothpaste, but were worried about its concentration in regular toothpaste. However, the main reason for most parents for using children’s toothpaste was safety if swallowed and taste. A mother thought that children’s varieties were made safe to swallow:

“Kids' toothpastes are safer if they swallow it.”

Regarding taste, parents commented about children’s toothpastes being “sweet” or “with appealing taste” and reported their child’s dislike of regular toothpastes as they found them “pungent” and unacceptable, especially if the parent used a type with mint or menthol.

Practices related to toothpaste amount, supervision, and spitting: After teaching their children how to brush, most parents supervised them for a brief period, followed by occasional supervision, even for very young children, and mainly on the toothpaste amount. A father spoke of the occasional lack of supervision on the toothpaste amount his son uses:

*“His mother puts little toothpaste for him. Sometimes he sees me and puts little toothpaste for himself, sometimes he fills the toothbrush.”* 

Nevertheless, most parents knew they needed to use a small amount of toothpaste, and few described the amount to be "pea size"or "smear" according to the child’s age. One parent thought a large amount of toothpaste would be better, and another said his child applied the same amount he saw in televised commercials. Regarding practices after brushing, most parents taught their children to gargle after brushing.

Challenges and efforts: Most parents who started children’s oral hygiene early indicated better acceptance for the routine and regularity compared to those who delayed it. The challenges reported to oral hygiene were pain during brushing, stubbornness, and refusal due to the toothpaste’s taste. Not all parents addressed these issues, which resulted in discontinuation of brushing. Parents who addressed the challenges tried assertiveness and persuasion, even though their methods did not always involve healthy alternatives. One of the mothers spoke about the challenges she faces with maintaining oral hygiene for her son and the methods she uses to overcome them:

“My son is very stubborn, and he does not agree (to brush). Sometimes I shout at him. Yes, sometimes he opens his mouth, sometimes he does not, so I tell him he will not go to the cold store, or ride in the car or go anywhere, so he starts brushing again." 

Knowledge, attitude, and practices related to children’s diet

Infants’ Diet

Knowledge and practices related to breastfeeding, bottle-feeding, pacifier use, and weaning were related in many cases to the family’s norms. Very few mothers had chosen to only breastfeed. As a benefit to breastfeeding, one mother noticed her children’s better health and immunity to common childhood diseases. Most mothers used a mix of bottle-feeding and breastfeeding. Some breastfed for five to six months and then introduced the child or totally shifted to bottle-feeding, while some introduced bottle-feeding at birth. The few mothers who exclusively bottle-fed did so either because the child was hospitalized or the mother did not produce milk after delivery. The main and most cited reason by parents for introducing bottle-feeding was the perception that the mother did not produce enough milk to provide the infant with enough nutrition or that the infant did not have its fill, which in turn worried parents about the infant’s weight. Parents in general linked infants’ health to their weight, a mother who had come for her three-year-old child and had only breast-fed her children and had grandchildren as well, commented on this saying:

“I knew my children felt full when I breastfeed them, so why would I introduce a bottle […] My daughters also breastfeed their infants […] and when you see the child, you can notice their good weight.”

Other reasons for using bottle-feeding were breastfeeding being time-consuming, the mother’s responsibility at home, the number of children, and the convenience of using bottle-feeding, especially when outside. A mother with seven children whose age ranged between five months, and 12 years of age commented on breastfeeding being time consuming:

“There is no time to always breastfeed […] I feel she - gets hungrier after she became five months old, she needs more nutrition, and more time but I have other children to care for." 

Regarding weaning, it was noticeable that even when mothers stopped breastfeeding early, they continued bottle-feeding till infants reached two years of age. A few mothers exceeded that limit either due to working or because another infant in the family was bottle-feeding.

Knowledge and practices related to pacifiers were related in many instances to family norms. Less than half of the parents introduced it to deal with the infant’s whining or putting things in its mouth. Those who did not use it thought it was unhealthy and unhygienic. A mother commented on this point, saying:

*“I do not like pacifiers because they fall to the ground […] then another child picks it up and puts it in his or her mouth. Sometimes hair might get wound around it”*.

Other parents feared that the use of pacifiers might become a prolonged habit with the child or might affect their teeth. A mother voiced her annoyance with the constant sucking sound of a pacifier.

General Diet

Parents in general knew that sugary items, fast foods, crisps, and soft drinks are unhealthy and noticed that after consuming such items, children either did not eat properly or abstained from eating homemade food. A mother spoke about her children’s food preferences even though she knew such choices were not always healthy:

*“My children eat items from the cold store [….] they do not eat homemade food, but he - son’s name- sometimes drinks milk.”* 

Many parents tried to include milk as a part of their children’s diet, and when they were not successful, they opted for flavored milk. Breakfast for many children consisted of cereals, sometimes chocolate-flavored. Parents understood the importance of a healthy diet for general health, which was evident when parents wanted to manage their weight or that of a child. Those who wanted to reduce a child’s weight to be healthier ensured that the child felt full but restricted the frequency and quantity of buying and consuming unhealthy items, including sugary foods and drinks, changed their food preparation habits, and increased consumption of fresh fruits and vegetables. Making dietary changes was not always easy for parents; a mother who wanted to lose weight and changed her food preparation methods likened it to “making hospital food”. Also, the changes were only made by the concerned member, parent with diabetes, or one who needed to lose weight, but not the whole family.

 Consumption of Sugary Foods and Drinks

Parents introduced sweetened foods and drinks early in life to their children and recognized their children’s high intake of such items. Foods high in sugar were, in many instances, consumed between meals. 

Regarding drinks, many parents said they controlled children’s intake of fizzy and soft drinks, but not juices and flavored milk. Parents either did not know or were not sure about the free sugar content in flavored milk, and more than half of parents reported their children’s regular intake of it. Regarding juices, there was also general confusion and a lack of knowledge regarding their free sugar content. Labels on juice packets and containers (juice, drink, natural, nectar, etc.) were confusing and misleading and led some to think that one type might be better than the other or contained less sugar, as in the case of a father who spoke about the type of drinks he buys for his children thinking that he buys the better option:

“*My children drink juices, not juice drinks.”* 

Regardless of parents’ beliefs and knowledge, most parents included juice as an integral part of their children’s regular meals at school and preschool, and sometimes considered juices part of a healthy diet. Many parents thought substituting juices was difficult, and alternative options were governed by children’s preferences. A mother spoke about this issue, saying:

“My children drink juice with every meal, and if there’s no juice, they won’t eat.”

Another mother explained that juice was important to add to her children’s diet being a liquid:

*“* *I do not think of juices in term of their sugars content, I look at them as liquids. The problem is that my children forget to drink water […] that is why I find it easier that they drink juice.”*

Many parents gave their children 150-200 ml juice containers, depending on their consumption, and many times the size or the number of containers increased with the child’s age and their preference, which in turn increased the consumed sugar amount. A mother spoke about her son’s preference in juice packet sizes:

“He asks me why I give him a small size juice packet (150 ml); he wants the bigger one, he says ‘I am not a small child now’.”

Factors Affecting Children’s Intake of Free Sugars

Parents recognized many factors that influenced their children’s access to and consumption of free sugars. 

Surrounding environment and commercial factors: Parents indicated that sugary foods are sold at schools’ canteens and used as reinforcement in preschools. Many parents also spoke about cold stores being close to their living areas. Such stores lack healthy items, while unhealthy items are available, varied, and cheap, and are more appealing to children. A father spoke of his children’s habit of going to the cold store and his dilemma with what to buy them because *“you do not get healthy options at the cold store.” *Another parent, a mother, spoke about the placement of sugary items at payment points in big supermarkets and how it attracts children:

“They place these large colorful lollipops at the cashier, and […] children get attracted to them and then they want them. They should place them somewhere else.”

Large family/family circumstances: Family circumstances affected breastfeeding practices and the use of pacifiers. Regarding diet, parents who live in joint families said it was not always possible to control their children’s intake of sugary items even if they have a separate living quarter or room and take their meals separately. Their reasons were that it is culturally unacceptable to refuse edibles from family members, especially elders, and that sugary items were invariably available at grandparents’ home, especially with other family members visiting. That also applied to working mothers who needed to leave their children under their grandparents’ care. A mother who lived in a flat with her in-laws spoke about the difficulty she faces with controlling her daughter’s diet since she goes down to her grandparents daily:

“We live with my husband’s family. My daughter meets with her cousins every day […] their mother is a nurse, and she brings them every day, and they bring sweets with them. Their aunt and their grandmother also give them sweets. My husband says I should not say no if they give our daughter anything." 

Differences between parents: Many times, the parent who accompanied the child for the appointment blamed the other for the child’s high sugar consumption or for not supporting dietary changes needed. Differences in dietary habits between mother’s and father’s families were mentioned by those who live in joint families, a mother spoke of differences between her family and her in-laws:

“My parents do not like children to eat the stuff sold at the cold store, but their father’s side are different; they say let the children go to the cold store and buy what they want.”

Buying sugary items in bulk: Many parents said they buy sugary foods and drinks in bulk. A mother described it as “*bringing the cold store home”*, rendering sugary items available to children all the time, free of charge and without needing permission or escort. Many times, the reason for this habit was to include such items in children’s lunchboxes or to use them as snacks or pacifiers at home. Some parents said they do so to stop children from buying at the schools’ canteens or going daily to the cold store, but in the end provide them with similar options such as *“chocolate sandwich, cupcake, juice, and popcorn.”*

Child’s nature: Most parents implied that it was their child’s nature to like and consume sugary items and that children were affected by their peers’ and friends’ dietary choices. Some parents spoke about children being stubborn and causing disturbance at home when they are not given their choice of snacks. A mother spoke about her son putting her under pressure with his demands of snacking:

“My son sometimes says I am a bad mother because I do not allow him to buy all the snacks he wants.”

Parents also considered children’s acceptance and preference when they contemplated changing dietary habits for the family.

Fear of depriving children: Many parents feared depriving their children if they forbade them from consuming food or drink that they had already seen at home or in the market. A mother spoke about this point when she explained her dilemma when her children go with her to the supermarket:

“Parents might give in and buy what their children see, so they do not feel deprived.”

That notion was also raised regarding providing similar amounts of pocket money, or comparable items to what other children had at school or with other children in joint families. A mother feared a negative impact on her children’s school performance if they felt deprived:

“Children look at each other in school. I try to explain to my son that different people might have different options, some more than the rest. But if I tell him ‘No’, his achievement at school gets affected. It happened before, and I do not want that.”

Knowledge, attitude, and practices related to childhood caries

To describe caries, many parents used various words such as “decayed”, “eroded”, “eaten up”, and “dissolved”.

Perceptions About the Cause of Caries

It was common knowledge to parents that “*sweets*” caused caries, and many noticed the difference between their children’s decay experience according to their sweets intake. Parents differentiated between types of sugary foods (*sweets, chocolates ... etc*.). One parent thought chocolates caused less caries than sweets. Parents did not recognize the cariogenicity of multiple sugar-containing items such as juices, ice cream, or sweetened cereals. However, parents recognized that late start of brushing and irregularity, sleeping without brushing, and sleeping witha feeding bottle in the mouth may cause caries. A mother spoke about the reasons she thought had resulted in her daughter getting caries at a young age:

“My daughter had decay because of eating sweets, she had also taken iron supplements […] and she would sleep with the bottle in her mouth.”

Some parents considered other factors such as weak build of the child (especially those born immature), caries being a family trait, intake of iron supplements, eating crisps, high intake of acidic foods and drinks, cracking nuts, nail biting, varying temperatures of ingested fluids, and types of saliva and teeth.

Reason for the Child’s First Dental Visit

Pain: Parents noticed that caries affected children’s appearance or caused halitosis, but most parents brought their child for their first dental visit due to pain, especially at night, or pain while eating, or inability to eat, or due to a swelling in the oral cavity or face. Few parents used alleviating medicines before seeking treatment. Noticing decay alone without a child’s complaints was not considered a reason to seek treatment for some parents, even when another child had dental complaints. Few parents learned about their children’s need for treatment after the educational institutes’ preadmission examination.

Effect of decay on child and family: The most troublesome effect cited by most parents was dental pain at night. It disturbed sleep for the child and parents, and affected the whole family’s schedule. Parents also felt helpless, tired, and sometimes had to seek private dental clinics. A mother spoke about her experience when her son used to get up at night with dental pain:

“It was tiring […] my son getting up at night with pain and would not go back to sleep.”

Parents noticed that tooth pain affected children’s quality of life and general daily behavior. A father spoke about his daughter’s suffering due to pain and the effects it had on her and the whole family:

“Yes, teeth ache affected her. My daughter was terribly upset, she did not want to do anything, and that upset us as well.”

Parents’ Efforts to Prevent and Control Childhood Caries

Changing habits: Many parents indicated that after a child suffered from dental pain, they made or tried to make changes such as abstaining from buying sugary items in bulk, or hiding them from sight, and reducing the amount or size of sweetened items. Some tried swapping unhealthy snacks for fruits and vegetables. A father spoke of the changes they made after his daughter suffered with dental pain:

“We used to buy packets of sweets and snacks and put them on the table, but after my daughter developed decay (and had pain), we stopped.”

Parents also spoke about the different challenges they faced in trying to make dietary changes, such as a lack of knowledge of better alternatives, the need for multiple and continuous efforts, and the need to adopt different parenting skills. Parents’ attempts at change were affected by children’s preferences and nature, and differences between parents or other family members in joint families. Many parents used the fear of pain to persuade children to reduce sugar consumption and maintain oral hygiene. A father spoke of how his daughter’s experience with dental pain had propelled her and her sibling to accept the dietary changes the family made:

“After my daughter had teeth pain, she accepted the changes we made for her diet […] even my other daughter after seeing her sister suffering with pain accepted the changes we made." 

Inquiries about fluoride varnish: Regardless of misinformation about fluoridated toothpastes, some parents showed interest in fluoride varnish application. They learned about it either through the history of application in the family, or from family friends, or from private clinics’ advertisements, as one father mentioned:

“I saw ads about the protection layer. I saw that it is placed on teeth early to prevent decay.”

Parents’ suggestions for improving services: More than one parent recognized the need for early prevention and detection of caries in children to ensure better prognosis. They recognized the opportunity and benefit of linking such services with the vaccination program provided by the Maternal and Child Health (MCH) services at PHCC as one father clearly indicated:

“We have children’s appointment at the MCH, why not allocate something similar for the child with the dentist [...] to give us advice on what to do, or find if the child has decay and then treat it from the beginning.”

Other suggestions included child-oriented dental clinics’ setup, trained staff in managing children’s anxiety, and provision of awareness sessions, especially about a healthy diet.

Sources of information

Parents mentioned several sources of information, namely, medical and dental health workers, social media, family members or friends, and advertisements. Many parents searched the internet and preferred YouTube (Alphabet Inc., Mountain View, California, United States) for ease of demonstration to children. With that, they sometimes adopted practices that differed from cultural norms. However, the main problem that parents faced with social media was the confusing and contradictory information, especially regarding fluoridated toothpastes. A mother who stopped using children’s variety of toothpaste around seven months back commented about doubting herself on this choice after seeing different information on social media:

“I stopped buying children’s toothpaste, but you know how social media is, it makes me doubt myself." 

Few parents reported receiving some information from medical health workers about breastfeeding, but limited or no information about formula milk and weaning age. Few parents reported receiving oral health care advice from dental practitioners, but it was sometimes varied, inconsistent, incomplete, or contradictory.

Children’s involvement in conversation 

Children who were six to nine years of age were seen to remind their parents many times of information related to their dietary habits. It was also noticed that parents sometimes asked the dentist to advise the child directly, regardless of their age, stating that the child would better comply.

## Discussion

Qualitative studies aid in exploring and understanding various complex social and ethnic factors that affect and determine oral health and disease [19]. This is the first qualitative study in primary healthcare centers and in Bahrain to explore parents’ attitudes, perceptions, knowledge, and practices related to children’s oral health, especially early childhood caries, and the factors affecting them. This is of importance given the high prevalence of caries in children in Bahrain. Lack of parental knowledge about fluorides, late introduction to brushing, unsupervised brushing, and difficult children are associated with early childhood caries [1], and all were seen in this study.

There was a noticeable culture of awareness about health and a positive attitude towards maintaining oral health. Many parents considered oral hygiene a part of daily life. This is in line with a cross-sectional sample of Bahraini mothers [20] and other qualitative studies from other regions [8,21]. This, however, differed from Alshammari et al. (2021), whose study sample may not be representative as it consisted only of fathers and male teachers [22].

Many parents started brushing their children’s teeth early, yet they missed starting at the recommended age. Also, the recommended age for supervision was not seen to be followed in many similar studies [6,8,9,21]. This differed from Sivaramakrishnan et al. (2022), who reported that only 33.7% of Bahraini mothers left children unsupervised [20]. This can be attributed to their questionnaire not including occasional supervision, which was reported by many parents in this group, for perceived safety related to the toothpaste amount. This lack of supervision on brushing may have caused the loss of the opportunity to prevent or notice disease early.

Challenges with brushing children’s teeth were reported by other studies [3,7,21]. The challenges in this sample were seen more in families with multiple young children. Irregular brushing was related to a delay in starting to brush till children’s enrollment in educational institutions, which is an important cornerstone of children’s life and stresses the need to use this period as a motivating factor and an opportunity for oral health promotion [23]. To overcome challenges with brushing, parents used incentives or modeling in similarity to other studies [2,8,21].

Most parents used children’s variants of toothpastes, as also reported by Sivaramakrishnan et al. (2022) [20]; such variants may not contain the effective fluoride concentration for caries prevention. Parents’ choice was driven by a lack of appropriate information about fluorides, children’s acceptance, and parents’ perception of the safety of toothpastes if swallowed, similar to other studies [6,8]. The availability of several types of toothpastes marketed to children with appealing packaging added to parents’ confusion. Some parents used comparatively expensive toothpaste, and none of them mentioned a lack or difficulty in obtaining toothpaste and toothbrushes, which could indicate their availability and affordability.

Traditional dietary habits have changed over time in Bahrain. However, parents understood what a healthy diet is, and although it was not voiced explicitly, the term "healthy food" was confusing to some. Early nutrition is detrimental for early childhood caries. Moreover, sugar was not clearly recognized as a common risk factor for diabetes, obesity, and dental caries. This was evident as the need to reduce sugar intake to control diabetes for a member did not involve the whole family.

Parents’ choice between breastfeeding and bottle-feeding affects children’s perception of taste and their future food acceptance [24]. In Bahrain, in 2020, only 12.6% of children under six months of age were exclusively breastfed [10], and 44% of mothers started complementary feeding before that [25]. Findings from this sample were similar and showed that breastfeeding was affected by misguided perceptions, family norms, and mothers’ responsibility at home. Also, the use of pacifiers might have interfered with breastfeeding for some. Bottle-feeding, which is known to be related to early childhood caries, was introduced early and stopped later than the recommended limit of 12 months of age. This might stem from mothers perceiving breastfeeding and bottle-feeding to be the same, hence weaning the child at two years of age, which is a cultural practice.

The high and regular consumption of sugary drinks, especially juices at a young age, was very noticeable and has become a norm for many. Sweetened drinks are the primary source of consumed free sugars and interfere with the intake of nutritious food [26]. Low consumption of water and plain milk and high consumption of sugary drinks at an early age is associated with high caries experience and is indicative of parents’ poor dietary practices and lack of knowledge [1].

Achalu et al. (2019) mentioned financial constraints in the ability to buy sweetened foods or drinks [9], which was not mentioned by participants; however, they opted to buy sugary items in bulk, which is more economical than buying from cold stores or schools’ canteens.

Regarding parents’ knowledge about caries, parents did not recognize the cariogenicity of many items other than sweets, similar to Aljafari et al. (2014) [7], and Dutch parents with high socioeconomic status (SES) reported by Duijster et al. (2015) [21]. However, they considered irregular brushing, sleeping without brushing, built of child, habits, and genetics as other probable causes of caries, similar to other studies [8,21]. Some parents confused the stains caused by Iron supplements with caries, a finding that was also reported elsewhere [9]. This confusion might have affected parents’ perception of the need to control children’s diet. 

Culture, social, and environmental factors affect children’s oral health [2]. Many such factors were seen in this study as well as reported by others [9,21,22]. A factor that was seen in this study to have facilitated the availability of free sugars and children’s high consumption was parents’ buying and storing sweets in bulk at home. Also, factors related to commercial determinants [27] were seen to affect parents’ choice and practice regarding oral hygiene, choice of toothpaste, and diet.

Children’s food choices are affected by family and elder peers [24], and family support is socially important and is associated with better health in children [2]. In this study, it affected parents’ ability to control children’s consumption of sugar. This can be attributed to parents’ need to conform to culture [9]. Moreover, in Arabian culture, forbidding children to eat or drink from what they saw to be available is considered unacceptable and is seen as a form of deprivation. Alshammari et al. (2021) mentioned that fathers feared being unkind to refuse their children’s choices of sweetened items [22], but the notion of fear of deprivation was not mentioned explicitly, as it was here. This fear, coupled with worry that a child might not have had its fill, affected parents’ dietary choices right from infancy. Children’s nature and their preferences influence parenting behaviors [1] and affect their ability to control diet and brushing routine, which was seen in the current study and elsewhere [3,7,8,21].

Treatment of caries in this sample was seen to be delayed. The accessibility concluded by other studies [8,9] may not be considered here, as dental primary care is provided free of charge in PHCC under health coverage schemes. Parents generally understood the importance of maintaining primary teeth, yet noticing decay did not warrant a dental visit. This, in part, can be attributed to parents’ lack of knowledge about the effect of caries on children, and issues related to early loss of primary teeth, which in turn can be related to lack of knowledge about the eruption time of permanent successor teeth. Eigbobo & Obiajunwa (2016) reported that, in Nigeria, seeking professional help was mostly done to alleviate symptoms [28], which was also seen in the current study, as parents delayed their children’s dental treatment till after the onset of symptoms and the effect on children’s quality of life. This was also reported by others [9]. Delaying dental treatment can be considered for the 42.8% estimated prevalence of untreated caries of deciduous teeth in children aged one to nine years in Bahrain in 2019 [29]. Pain was also the main instigator for some parents to control their children’s diet and oral hygiene.

Parents in this group showed many variations in knowledge and practices in relation to childhood caries than what is advised based on evidence [23], yet they showed willingness and perception of their ability to control dental disease as well as other health issues in the family. Similar findings were reported by high SES Dutch participants, but not low SES parents and first-generation immigrant parents from Morocco and Turkey [21], and not the fathers’ sample reported by Alshammari et al. [22]. Parents also have a high acceptance of institutionalized prevention since they recognized the need for early detection and prevention of caries at a young age and recognized a facilitating factor in linking caries prevention and promotion programs to the MCH vaccination program at the PHCC. Parents also recognized the need for awareness sessions, which necessitate reducing variations between health practitioners in Bahrain [11,25]. Baghlaf (2023) questioned children’s participation in qualitative data [4], but their participation in conversations about diet is worth noticing.

Strengths and limitations

The strengths of this study include being the first qualitative study in Bahrain and in PHCC to explore parents’ attitudes, knowledge, and practices in relation to childhood caries. The study provided insight into parents’ knowledge and practices and the different environmental and cultural factors that affected them as early as infant feeding and the start of oral care for their children. The study was also able to draw an idea of parents’ practices and change over time, since the discussion included all their children, not only the child being treated, and hence possibly reducing bias recall.

The limitations of the study include having a small, convenience sample only from the northern governorate, which has the highest population and the largest number of households in Bahrain [30]; hence, although the study may represent a considerable proportion of parents in this governorate, it may not be generalizable to others. The sample size in quantitative studies is debatable, as it is determined when data saturation is attained, but it could be attained with a small number when the sample is homogenous, as in this study. Another limitation is the issue of having a one point of view from having a single researcher. To improve credibility and validate the results, mixed triangulation was done by comparing the results with other published qualitative studies from different regions, as well as available quantitative data from Bahrain, and when a difference was found, the plausible reason was discussed.

## Conclusions

The study explored qualitatively Bahraini parents’ attitudes, knowledge, and practices related to children’s oral health, especially early childhood caries, and shed light on the possible gaps in parents’ knowledge and practices. Several detrimental factors were noticed, such as early introduction and late weaning of bottle-feeding, early introduction of sugars, high consumption of sugary drinks, and a lack of knowledge about and delay in use of fluoridated toothpaste. All of these were affected by multiple cultural and environmental factors. The study provided an insight into the situation, but to formulate a clear picture of oral disease burden in children in Bahrain and the factors affecting this, including SES and the education level of parents, several population-wide quantitative and qualitative studies are needed.

## Appendices

**Table 4:** Semi-structured questions for interview

Topic	First line of questions	Exploratory questions
General information about parent and family	Age of parent	
	Working status	
	Number of children	Age of their children
General knowledge about primary teeth	Are primary teeth important?	Why?
	Age of eruption	
	Age of exfoliation	
	When do children finish changing their primary teeth to permanent teeth	Will it matter if a primary tooth is lost before its exfoliation time, why?
Care and hygiene of children’s teeth	If they brush their children’s teeth.	When did they start brushing their children’s teeth? Importance of brushing children’s teeth How did they start/ using what? How many times a day? Are they regular? If not regular, why? What are the difficulties?
	Use of toothpaste	What do they know about toothpastes? From where they know this information? Is it important to use a toothpaste and why? If they mention fluoride: what do you know about it? What is their source of information? What toothpaste do they use for their children? and why? What factors affect their use of toothpaste? At what age did they start using toothpaste and why? What is the toothpaste amount they use, and why? Who puts the toothpaste for the child? Who brushes the child’s teeth? Do they supervise children’s brushing? How often? At what age do they leave the child to brush alone?
	Practices after finishing brushing	What do they tutor the child to do? gargling or spitting?
	Factors – if any- that aided parents in children’s oral hygiene practices. Difficulties -if any- related to children’s oral hygiene practices	What were the difficulties if any? Did they address them? How? Were they successful? And if not, what was/were the cause/s?
fluorides	Have they ever heard about fluoride?	What do you know about it? From where? Fluoride in toothpaste? (If not discussed before). If fluoride varnish was mentioned: what do they know? From where did they hear/ learn about it? Did they seek it/ was it applied? If not, why?
Early child diet and habits	What was their children’s feeding as infants (breastfeeding/ bottle feeding)?	Why? When was bottle-feeding start, when did it stop? For how long was the feeding (breastfeeding and bottle-feeding) continued, and why? If Breastfeeding was stopped early, why, and what were the difficulties if any?
	Did they use Pacifiers?	Why whether yes or no? When did they stop it?
	Did they receive any information/ advice regarding breastfeeding/ bottle-feeding/ weaning/ pacifier use?	If yes, by whom? What was the advice?
	When were solid foods introduced?	What were the foods and drinks introduced, why?
General Diet	What does children eat for breakfast?	What types? If sweetened or not?
	Do you put drinks (any) with meals?	If any sweetened drink, what and why?
Dental visits	Child’s first dental visit	At what age?
	What was the reason of the visit?	Details of the reason
	If pain/ oral complains	What? How did they deal with it? Were they successful? How did the dental complain affect the child and the family?
	Dental visits of other children (other than the child being treated)	Did you bring your other children for a dental checkup? When was the first time? Why?
	In case of pain/ oral and dental complains of a child, did it propel the parents to bring other children for dental checkup?	If not, why?
	Were there any changes noticed in the family/ child after the experience?	What changes?
Cause of caries	What are the factors that might affect teeth/ oral health in children (according to parents)?	
	What do you think causes caries?	
	When parents talk about sugars/ sweets:	What are the items that contain sugars that their children consume? In general, what are the items that contain sugars?
Sugars Consumption	Where do children get their (sugary items i.e., sweets/ sugars) from? the follow-up exploratory questions depend on the answer/ answers At home	What are the sugary items available at home? How are these items available (buying habits of parents), Who buys them? Why? How frequent are these items available? How often does the children consume these items? How many do they consume?
	In joint families: From grandparents / relatives	Does the whole family take their meals together? What do they drink with their main meals? How many times a day does the child get sweet items (from relatives)? Do the parents have sugary items at their living space? What does the child take to the school/ Kindergarten?
	From the cold store	Why do/does the children/child go? How many times per day or per week does the children go? With whom does the children go? What do they buy? How many items? What packet size? Why? What is the allowance for each visit?
	At school/ Kindergarten	What do they (does he/ she) take in their lunch box? How many sugar-containing items? What is the packet size? Why? Do the children (does he/ she) take allowance to school? What do they (he/she) buy?
	What are the factors that played a role in their children’s consumption of sugar	Depending on the factors/ discussion are the follow-up questions.
Attempts at addressing the issue (reducing caries/ preventing caries/ reducing sugar intake?	Did the parents try to address these issues?	What did they do? Were they successful? How? If they were not successful, what were the difficulties they faced? Did they try to address them, and how?
Oral health advice/ Information	Did you receive any information or advice about oral health?	What was it? Who provided it? Were they able to follow the advice? If not, why?
Advice/ suggestions to services	What?	How to achieve it?
Further information	Anything the parent wants to add that was not discussed.	
